# An overview of the nutritional status of childbearing age women, children and adolescents living in a rural area of Madagascar: preliminary results of the Tany Vao project

**DOI:** 10.1017/S1368980024000259

**Published:** 2024-01-29

**Authors:** Maria Vittori Conti, Leila Itani, Alice Beretta, Kassandra Yaghi, Asia Filosa, Cristina Monti, Hellas Cena

**Affiliations:** 1 Laboratory of Dietetics and Clinical Nutrition, Department of Public Health, Experimental and Forensic Medicine, University of Pavia, Via Bassi 21, 27100, Pavia, Italy; 2 Department of Nutrition and Dietetics, Faculty of Health Sciences, Beirut Arab University, Beirut 11072809, Lebanon; 3 Department of Public Health Experimental and Forensic Medicine, Unit of Biostatistics and Clinical Epidemiology, University of Pavia, Pavia, Italy; 4 Clinical Nutrition and Dietetics Service, Unit of Internal Medicine and Endocrinology, ICS Maugeri IRCCS, 27100 Pavia, Italy

**Keywords:** Public health, Madagascar, Nutrition

## Abstract

**Objective:**

To describe the food consumption, nutrition knowledge and nutritional assessment of childbearing age women and their children, living in rural villages in Madagascar. The results presented are related to the Tany Vao research study.

**Design:**

A cross-sectional pilot study.

**Setting:**

The study was carried out in Ampanitosoha village on Nosy Mitsio island in Madagascar.

**Participants:**

32 women (14–49 years) and 36 children and adolescents (2–17 years).

**Results:**

70 % of the women lacked nutrition knowledge and did not reach the Minimum Dietary Diversity Index for Women cut-off. The median BMI was 21·1 kg/m^2^ but 55·2 % of the women exceeded the cut-off for waist-to-hip ratio, 51·7 % for waist-to-height ratio and 81·2 % for mid-upper arm circumference (MUAC). Almost all had adequate intake of energy, protein and carbohydrates, while 27·6 % had excessive fat intake and 75·9 % of added sugars. Over half of the women did not meet the micronutrients Reference Daily Intake (RDI). For children, the MUAC z-score was lower for boys than for girls (*P*-value = 0·041).

**Conclusions:**

These results underline the importance of increasing women’s nutritional knowledge to promote healthy pregnancy and lactation. Moreover, it is fundamental to provide people living in rural areas with sustainable tools to improve dietary diversity and support long-term health.

The WHO stated the need for global action to reduce undernutrition by 40 % by 2025 (SDGs#2)^(1)^. Yet, progress made worldwide by most countries to decrease malnutrition in its three forms (overnutrition, undernutrition and micronutrient-related malnutrition) cannot meet the global targets by 2025^(2)^. Of particular importance are low-income countries where maternal and child malnutrition have been reported to be still unacceptably highly prevalent^(3)^.

Internationally, while about 2·2 billion adults are either overweight or affected by obesity, around 38·9 million children under five are overweight, 149·2 million are stunted and 45·5 million are wasted^(2)^. Notably, low-income countries host 99 % of children with malnutrition^(4)^. UNICEF-WHO-World Bank Group Joint Malnutrition 2021 Estimates showed that two out of five stunted children lived in Sub-Saharan Africa in 2020^(2)^.

Micronutrient deficiencies also are still prevalent globally among women and children. Around 372 million (56 %) preschool children and 1·2 billion (69 %) women of childbearing age (14–49 years) are estimated to have micronutrient deficiency^(5)^. The highest prevalence was observed in Sub-Saharan Africa reaching 62 % among preschool children and 80 % among women of childbearing age^(5)^. Micronutrient deficiency, including iron, zinc, iodine and vitamin A deficiency, are prevalent in low- and middle-income countries due to inadequate complementary foods and infectious diseases such as diarrhoea^(3)^. Furthermore, anaemia among women of childbearing age remains of course the global targets in most countries affecting 570·8 million girls and women^(2)^.

Within this context, Madagascar is one of the countries where the prevalence of malnutrition still represents an emergency, particularly in rural areas^(6)^. Considering global nutrition targets, Madagascar showed either no progress or worsening towards the global targets for breast-feeding (50·6 %) and anaemia among women 14–49 years (37·8 %). Some progress was reported for stunting (39·1 %), wasting (7·7 %) and low birth weight in 2015 (17·1 %)^(2)^. Some progress on the course of childhood overweight and obesity and of women obesity were also reported (9·2 %). Madagascar also was reported to show a decrease in vitamin A deficiency between 1990 and 2019^(7)^ as well as iodine deficiency and protein-energy malnutrition^(8)^ although these conditions are still prevalent.

The state of malnutrition in Madagascar is directly reflected in the persistent trend of an alarming Global Hunger Index score between 2000 (42·7) and 2022 (38·7)^(9)^.

The score that characterises Madagascar highlights its impending emergency, since it is composed by considering the undernourishment condition, child stunting, wasting and mortality^(9)^. It is thus composed by referring to the most vulnerable segments of the population. The condition is paralleled by the acute food insecurity state affecting 33 % of people and led by several causes including persistent economic crises and instability, poverty, climate change with extreme weather conditions and drought affecting agricultural crops and income and limited health services^(9)^. Not to mention cultural aspects of food intake and eating habits and geographic in-accessibility^(10)^.

This situation represents a salient public health problem given the impact on vulnerable population groups, namely women, infants and children. The health and nutritional status of a female during her own foetal life, infancy and periconceptional period, determines the nature of her pregnancy outcome in terms of herself short and long-term health as well as that of her offspring^(11)^. Not to mention the adverse impact of nutritional deficiencies on growth, development, cognitive and physical performance or economic productivity^(12)^ which can transcend to future generations^(13)^. Given the developmental plasticity of early life^(11)^, and the intergenerational impact of adverse nutrition exposures, the first 1000 d are a critical window for interventions through reformation, conservation and efficient use of local environmental^(6)^. In Madagascar, the great variety and quantity of natural resources could represent a potential solution to improve the health condition of the local population, especially for those living in rural areas with low access to food supplies^(10,14)^.

The ‘Tany Vao’ (meaning ‘new land’ in Malagasy language) is a multidisciplinary project conducted by four teams, including nutritionists (represented by Laboratorio di Dietetica e Nutrizione Clinica), environmental engineers and agronomists (represented by Kukula NGO; https://kukula.it/tany-vao/), medical doctors (represented by Nave Ospedale Elpis) and engineers specialised in water, sanitation and hygiene (represented by Help for Optimism NGO, principal investigation; https://helpforoptimism.org/projects/nutrition-programme/). The main objective is to improve the health status of populations living in the Mitsio Islands (rural area of Madagascar) through the development of a multidisciplinary and sustainable intervention model.

This manuscript presents the results of the cross-sectional pilot study of the nutrition team that will be used as a starting point for the future interventional part of the Tany Vao project.

## Materials and methods

### Study design and setting

A cross-sectional pilot study was conducted to describe and evaluate nutrition conditions of the women of childbearing age (14–49 years old) and their children living in rural areas of Madagascar. The study was undertaken on the Nosy Mitsio island, 20 miles away from Madagascar. Specifically, recruitment was conducted in Grand Mitsio, the already large and inhabited island of the Mitsio Islands archipelago. The population is divided into several fishing villages – Ampanitsoha, Ratapenjiki, Ambarimidada, Bevaoko, Ampasindava and Marimbe. For each village, the researchers with a local cultural mediator support asked the village chief if they would like to participate in the Tany Vao study, explaining to them the objectives and benefits. The villages that joined were those of Bevono and Ampanitsoha, and in this paper, the results for the village of Ampanitsoha are presented.

Ampanitosoha village is characterised by a lack of essential services and infrastructure, such as access to clean water, hospitals and food storage areas. For this reason, the population is highly vulnerable especially with regard to health and nutritional status.

The cross-sectional phase took place between the beginning of April and the end of May 2022. Data collection was carried out in the Ampanitosoha school, where the researchers established a work setting.

All enrolled participants signed an informed consent form, translated in Malagasy language. For individuals under the age of 18, the informed consent form was signed by one of the parents or a caregiver. This study was conducted according to the guidelines laid down in the Declaration of Helsinki.

Study approval was granted by the Help For Optimism NGO, located in Hell-Ville city (Nosy Be, Madagascar). To facilitate research, the President of Help For Optimism NGO was empowered to issue research clearance to staff on behalf of the municipality of Hell-Ville within the Tany Vao project, funded by Chiesa evangelica Valdese e Fondazione Istituto Vismara De Petri Onlus.

### Tany Vao project description

The Tany Vao project aims to establish a circular and sustainable intervention model to enhance the health condition of the population living in the Nosy Mitsio Villages. The main deliverable of the project is the structuring of vegetable gardens and the creation of an irrigation system. This system is designed to serve as a sustainable livelihood tool for the local community, leveraging the natural resources available in the area – thus emphasising the crucial theme of sustainability. Given that the local population lacks a traditional background in agriculture, the Tany Vao project’s intervention can be characterised as ‘innovative’ within the context of its development. The implementation of the gardens and the use of the vegetable products by the local population will contribute to an increased intake of essential minerals and proteins, facilitated by the cultivation of fruit trees, a variety of vegetables, and legumes.

Each specialised team, comprising environmental engineers, agronomists, water, sanitation, and hygiene engineers, as well as nutritionists, conducted preliminary observations before the intervention phase. This meticulous approach served as the foundation for the project, particularly since the remote nature of the area meant that no pre-existing studies were available to comprehensively describe the prevailing situation. Specifically, as regards the nutritional part (on which this paper focuses), the authors decided to address the most vulnerable populations (therefore women of childbearing age and their children) having to make a selection due to the complexity of the territory and of resources available. The nutritional team decided to target women of childbearing age and their children. As regards the paper presented, it describes the results relating to the data collected and analysed in the observation phase of the Tany Vao project. Based on the results, respectively collected by each team, of the observational study the intervention will be structured.

### Participants, inclusion criteria and sampling

The study sample included women of childbearing age (14–49 years old), pregnant or not, who accepted to participate and signed the informed consent, and their male or female children/adolescents (2–18 years old) living in the Ampanitosoha village.

As this is a cross-sectional pilot study with a descriptive primary objective, the sample size was not calculated, all the eligible women in the village were considered for participation in the study. Data were collected from all eligible women and children in the village.

### Variables and data sources

The nutrition team of the Tany Vao project analysed the health status of the population from a nutritional point of view, through nutritional assessment, based on the collection of information such as nutritional knowledge, dietary intake, diet quality and anthropometric measurements. A more in-depth description of the variables collected and the methodology used is reported below.

#### Sociodemographic characteristics

Questions that retrieved information on age, religion, ethnicity, civil status and level of education were included in the sociodemographic characteristics section.

#### Nutrition knowledge

Nutrition knowledge was assessed using a Nutrition Knowledge Questionnaire. The authors started with a previously validated Nutrition Knowledge Questionnaire^(15)^ consisting of 29 questions and adapted it to the local population, based on their culture and educational level. The final version was a 6-question tool. The questions tackled mothers’ knowledge in terms of the best food to eat, the amount of water to drink and what practices are better to avoid during pregnancy, the best food for infants before 6 months of age, and where to find calcium and why it is important for health.

#### Dietary intake assessment

A semi-structured 24h recall (24-hR) was administered to women participating in the study to estimate energy and nutrient intake. In the absence of a validated reference for portion size, a photographic portion size atlas (specific to the Malagasy population) was developed to identify the quantity consumed by the respondent (the tool is available in supplementary materials). The portions described were based on most used household tools and the weight of commonly consumed foods.

Dietary intake data from the 24-hR were analysed for energy, macro- and micronutrient. In the absence of a food composition database for the Nosy Mitsio population, a specific one was created based on the food composition data of the U.S. Department of Agriculture-USDA^(16)^ and the Tanzanian Food Composition Tables^(17)^ (the authors chose Tanzania as one of the Sub-Saharan Africa countries where food composition tables are validated and accessible). The created food composition database provided data on energy (kcal), total proteins (g), total fats (g), saturated fats (g), cholesterol (mg), carbohydrates (g), sugar (g), fibre (g), vitamin A (µg), beta carotene (µg), vitamin E (mg), vitamin C (mg), vitamin B_12_ (µg), folate (µg), calcium (mg), iron (mg), zinc (mg), magnesium (mg) and sodium (mg).

#### Dietary quality assessment

Diet quality was evaluated with the Minimum Dietary Diversity Index for Women (MDD-W), a food group diversity indicator that has been shown to reflect one key dimension of diet quality: micronutrient adequacy, summarised across 11 micronutrients^(18)^. This is a dietary diversity indicator developed exclusively for women, because of nutritional vulnerability affecting them during pregnancy and lactation^(19)^. For all these reasons, the MDD-W^(18)^ was evaluated on women, based on 24h-R. According to the MDD-W protocol^(18)^, foods were classified into ten groups: (1) grains, white roots and tubers, and plantains; (2) pulses; (3) nuts and seeds; (4) dairy; (5) meat, poultry and fish; (6) eggs; (7) dark green leafy vegetables; (8) other vitamin A-rich fruits and vegetables; (9) other vegetables; (10) and other fruits. Food groups included in the MDD-W index mostly suggested diet quality with adequate micronutrient intake considering the most important micronutrients^(18)^. For each food group, a dichotomous variable was used: ‘1’ for women who consumed any food item in the group at least one time per day and ‘0’ for those who did not consume any food within that food group. The MDD-W index was calculated by summing up the number of food groups consumed. The adequacy in micronutrient intake was reached when the index was equal to or higher than 5^(18)^.

#### Anthropometric measurements

Concerning the women’s nutritional assessment, anthropometric measurements, including body weight (kg), height (cm), waist circumference (cm), arm circumference (cm) and triceps skinfold (mm), were collected. As for children, the nutritional assessment included body weight (kg), height (cm) and mid-upper arm circumference – MUAC (cm)^(20)^.

All measurements were done using standard techniques and equipment. Before any anthropometric assessments were taken, the research team asked the women to remove shoes and to dress in standard light clothes (sleeveless shirt and skirt) provided by the research team. As for children, their clothes and shoes were removed before any anthropometric measurements were taken. Mothers were asked to hold the children while standing on the electronic weighing scale, and the children’s body weight was taken by the difference method.

Body weight was measured using a portable scale to the nearest 100 g (Tanita HD-366 Digital Weight Scale). Height was measured to the nearest 1 mm using a portable altimeter (Seca 213 Portable Measuring Rod) with the subject standing erect with arms along the sides, weight evenly distributed on the feet forming a 60° angle with each other, and gaze turned toward the horizon^(21)^.

In the same standard condition, waist circumference was measured using a flexible yardstick at the midpoint between the lower margin of the least palpable rib and the top of the iliac crest.

MUAC was measured on the left arm using a specific insertion tape to the nearest 0·1 cm at the midpoint between the acromion process of the scapula and the olecranon process at the tip of the elbow (Abbott Laboratories Inc., Columbus, OH, USA).

Triceps skinfold thickness was measured to the nearest 1 mm using the ‘Lange’ brand callipers (Beta Technology, Santa Cruz, CA, USA).

Maternal BMI was computed using the standard formula by dividing weight in kilograms by the square of height in metres (kg/m^2^). Based on the WHO, BMI classification subjects were categorised as follows: BMI < 18·5 = underweight; 18·5 ≤ BMI < 25 = normal weight; 25 ≤ BMI < 30 overweight; BMI ≥ 30 obese. Other anthropometric indicators used for women also include waist-to-hip ratio, waist-to-height ratio and arm muscle area.

The WHO 2006 growth reference standards, which uses the WHO Multicentre Growth Reference Study population, were used to transform children’s measurements into sex- and age-specific z-scores: height-for-age z-score, weight-for-age z-score, BMI-for-age z-score and MUAC-for-age z-score. Moreover, according to the WHO, malnutrition is defined as stunting when height-for-age z-score is below –2sd, underweight when weight-for-age z-score is below –2sd, wasting when BMI-for-age z-score is below –2sd and diagnosis of malnutrition when mid-upper arm circumference -for-age z-score is below –2sd ^(22)^.

#### Data collection

All interviews were carried out in the Malagasy dialect by the same operator supported by local enumerators, previously trained. Data collection for NKQ and the 24-hR answers were done using the Open Data Kit application^(23)^, an open-source mobile data software program used to collect data quickly, accurately and offline. The data collected were kept in cloud storage and then transferred for statistical analysis. All the anthropometric data collected were entered using an offline laptop with the last version of Microsoft Excel.

### Statistical analysis

All statistical analyses were conducted using Stata software (version 17; StataCorp LP). Descriptive statistics for women and children were calculated: specifically, for quantitative variables that were normally distributed according to the Shapiro-Wilk test, mean and sd were reported; otherwise, median and interquartile range are used. In both cases, minimum and maximum values were computed. Whereas for categorical variables absolute and relative frequencies were calculated.

To compare the mean z-score for growth indices between boys and girls, Student’s *t* tests were computed, whereas in case normality was not satisfied Wilcoxon–Mann–Whitney test was performed.

## Results

A total of 32 women of childbearing age (14–49 years) and 36 children and adolescents (1–17 years), including 15 girls and 21 boys, were enrolled in the Tany Vao pilot study.

### Women

The median age of the women was 23·0 (interquartile range: 20·0–34·0) years ranging between 17·0 and 45·0 years. Table 1 presents the sociodemographic characteristics of only 31 women, as one was not available to answer the questionnaire. All the participants (100 %) belonged to the Sakalava Antakatagna ethnicity with the majority being Muslims (87·1 %), married (64·5 %) and mostly having a primary education (35·5 %) followed by secondary education (29·0 %) or no education (25·8 %), with the least having high school education (9·7 %).

**Table 1:** Sociodemographic characteristics of the women (*n* 31)

Variable	*n*	%
Religion		
Muslim	27	87·1
Atheist	4	12·9
Civil status		
Married	20	64·5
Divorced	7	22·6
Single	4	12·9
Education level		
No education	8	25·8
Primary school	11	35·5
Secondary school	9	29·0
High school	3	9·7

Description of sociodemographic characteristics of women population (*n* 31). For categorical variables, absolute and relative frequencies were reported *n* (%).

Table 2 presents the results of the nutrition knowledge assessment. Tany Vao’s female population lacks nutritional knowledge: more than 70 % answered incorrectly about dietary sources of calcium (87·1 %), its importance to human health (71·0 %) and complementary food sources (96·8 %). On healthy eating behaviour during pregnancy, the majority answered correctly (90·3 %).

**Table 2:** Nutrition knowledge of the women (*n* 31)

Variable	*n*	%
Which are the food rich in Calcium		
Wrong	27	87·1
Correct	4	12·9
Why is calcium intake important for human health?		
Wrong	22	71·0
Correct	9	29·0
What kind of behaviours/ practices are better to avoid during pregnancy?		
Wrong	3	9·7
Correct	28	90·3
What is the best feeding source for infants before 6 months of age?		
Wrong	30	96·8
Correct	1	3·2
How many litres of water shall pregnant women drink?		
Wrong	25	80·6
Correct	6	19·4

Description of nutritional knowledge of women population (*n* 31).The absolute and relative frequencies of correct answers were reported (*n* (%)).

Table 3 shows the means and sd or median and interquartile ranges for the dietary intake of energy, macronutrients and micronutrients.

**Table 3:** Energy, macro- and micronutrient intake of women (*n* 32)

Energy, macro- and micronutrient intake												
	Mean	sd	Median	25th–75th	Min–Max	Inadequate		Adequate		Excess		Ref
						*n*	%	*n*	%	*n*	%	
Energy (kcal)	3265·9	951·3	–		982·3–5096·4	2	6·9	27	93·1	–		^(23,24)^
Total protein (g)	97·7	31·6	–		38·8–163·1	1	3·4	28	96·6	–		^(25)^
Total fat (g)	–		49·6	30·1–89·3	7·7–170·8	14	48·3	7	24·1	8	27·6	^(25)^
Saturated fat (g)	–		11·5	5·1–27·9	1·4–135·2	–		21	72·4	8	27·6	^(26)^
Cholesterol (mg)	167·0	113·9	–		0·0–522·5	–		29	90·6	3	9·4	^(27)^
Cho (g)	567·7	168·8	–		179·5–1023·1	–		32	100	–		^(25)^
Sugar (g)	–		45·1	17·5–85·1	2·5–240·3	–		28	96·6	1	3·4	^(28)^
Added sugar (g)	–		11·7	0·0–51·8	0·0–195·7	–		7	24·1	22	75·9	^(28)^
Fibre (g)	–		12·5	9·6–15·6	4·8–42·2	30	93·9	2	6·1	–		^(25)^
Vitamin A (µg)	–		377·8	247·8–490·6	37·0–7633·2	25	78·1	7	21·9	–		^(29)^
Beta-carotene (µg)	–		70·3	23·0–833·9	0·0–13 144·3	18	56·3	14	43·7	–		^(29)^
Vitamin E (mg)	–		13·3	7·3–19·6	2·9–52·7	22	68·8	10	31·2	–		^(29)^
Vitamin C (mg)	–		48·7	34·6–71·2	2·8–340·9	14	43·7	18	56·3	–		^(29)^
Vitamin B_12_ (µg)	–		5·3	2·4–10·6	0·0–67·3	8	25	24	75	–		^(29)^
Folate (µg)	–		162·7	117·7–220·7	47·4–916·3	30	93·8	2	6·2	–		^(29)^
Calcium (mg)	–		318·6	252·6–382·9	135·4–1421·2	31	96·9	1	3·1	–		^(29)^
Iron (mg)	–		11·1	8·0–14·2	4·5–28·4	29	90·6	3	9·4	–		^(29)^
Zinc (mg)	11·5	4·3	–		3·6–21·7	16	50	16	50	–		^(29)^
Magnesium (mg)	–		339·1	271·5–423·7	137·5–842·1	3	9·4	29	90·6	–		^(29)^
Sodium (mg)	–		1584·2	524·6–2647·9	117·3–5013·8	–		18	56·2	14	43·8	^(29,30)^

Description of energy, macro- and micronutrients intake of women population (*n* 32). According to quantitative variables’ distributions, mean and sd or median and interquartile range were reported with minimum and maximum values. Additionally, cut-off points for adequacy were based on age-specific reference intake values taking into consideration the physiologic condition of being pregnant or lactating. Frequencies and percentages are reported as inadequate (below the cut-off), adequate (within the reference range) or excess (above the cut-off) intake. Reference cut-offs were calculated/evaluated as reported in supplementary materials (online Supplementary Table S1).

The participants consumed on average 3265·9 ± 951·3 kcal/d with a minimum intake of 982·3 and maximum intake of 5096·4 kcal/d with 93·1 % having an adequate energy intake. In terms of macronutrient consumption, the majority (96·6 %) of the population satisfied with protein intake and carbohydrate intakes (100 %). Regarding total fat intake, while only 24·1 % of the population consumes it in adequate amounts, nearly half of the participating women (48·3 %) had inadequate intake and 27·6 % had excessive intake. As for simple carbohydrates, ‘sugar’ and ‘added sugar’ were reported separately. For sugar intake, 96·6 % of the population had an adequate intake; in contrast, 75·9 % of the population exceeded the allowance for added sugars. Fibre intake was not adequate for 93·9 % of the participating women, with a median intake of 12·5 g/d.

Considering micronutrients, over half of the participating women did not meet the recommended daily intake. Micronutrients that were in short supply included vitamin A (78·1 %), *β*-carotene (56·3 %), vitamin E (68·8 %), folate (93·8 %), calcium (96·6 %), iron (90·6 %) and zinc (50·0 %). As for sodium intake, it was above the daily allowance for 43·8 % of the participating women.

**Fig. 1:**
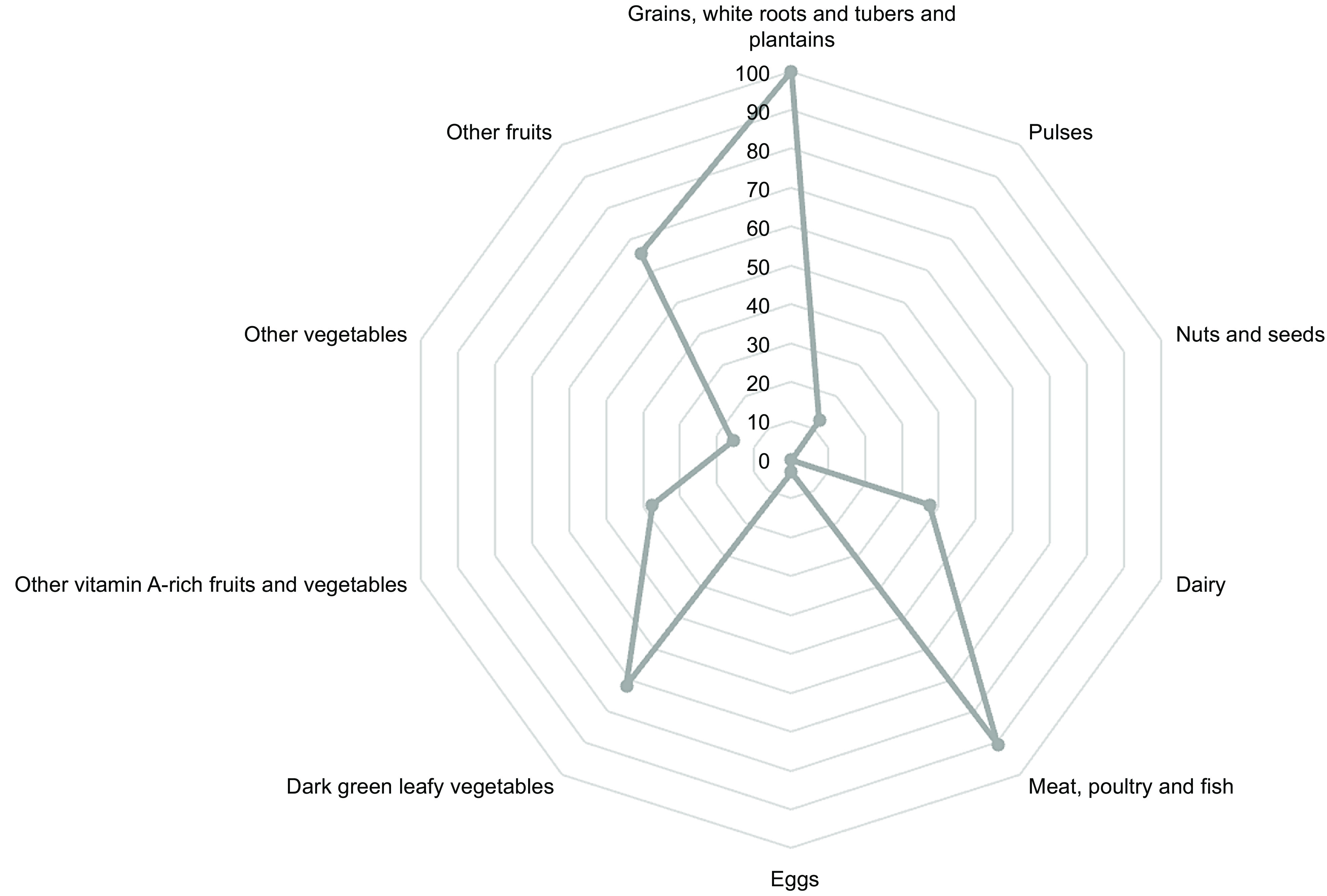
Number of women reporting consumption of the 10 different food groups considered to evaluate the minimum dietary diversity index for women (MDDI-W) reported as percentage

The micronutrient intake deficiency was reflected in the women’s dietary diversity index. Total mean MDD-W index was 4·3 and micronutrient adequacy (MDD-W index ≥ 5)^(15,18)^ was achieved only by 10 women constituting 31·2% of the whole sample. Table 4 shows the reported consumption of different food groups that were used for the MDD-W index calculation. The most frequently consumed groups were ‘white roots and tubers and plantains’ (100%), followed by meat, poultry and fish (90·6%), dark green leafy vegetable (71·9%), other fruits (65·6%) and other vitamin A-rich fruits and vegetables and dairy (37·5%). While ‘nuts and seeds’ (0 %), ‘eggs’ (3·1%) and ‘other vegetables’ (15·5%) were least frequently consumed (Fig. 1).

**Table 4:** Number of women consuming the different food groups in the MDD-W (*n* 32)

Food groups consumed	*n*	%
Grains, white roots and tubers, and plantains	32	100
Pulses	4	12·5
Nuts and seeds	0	0
Dairy	12	37·5
Meat, poultry and fish	29	90·6
Eggs	1	3·1
Dark green leafy vegetables	23	71·9
Other vitamin A-rich fruits and vegetables	12	37·5
Other vegetables	5	15·6
Other fruits	21	65·6

Description in absolute frequencies (*n*) and percentage (%) of the women (*n* 32) consuming the 10 different food groups considered to evaluate the Minimum Dietary Diversity Index for Women (MDDI-W)^(18)^.

**Fig. 2:**
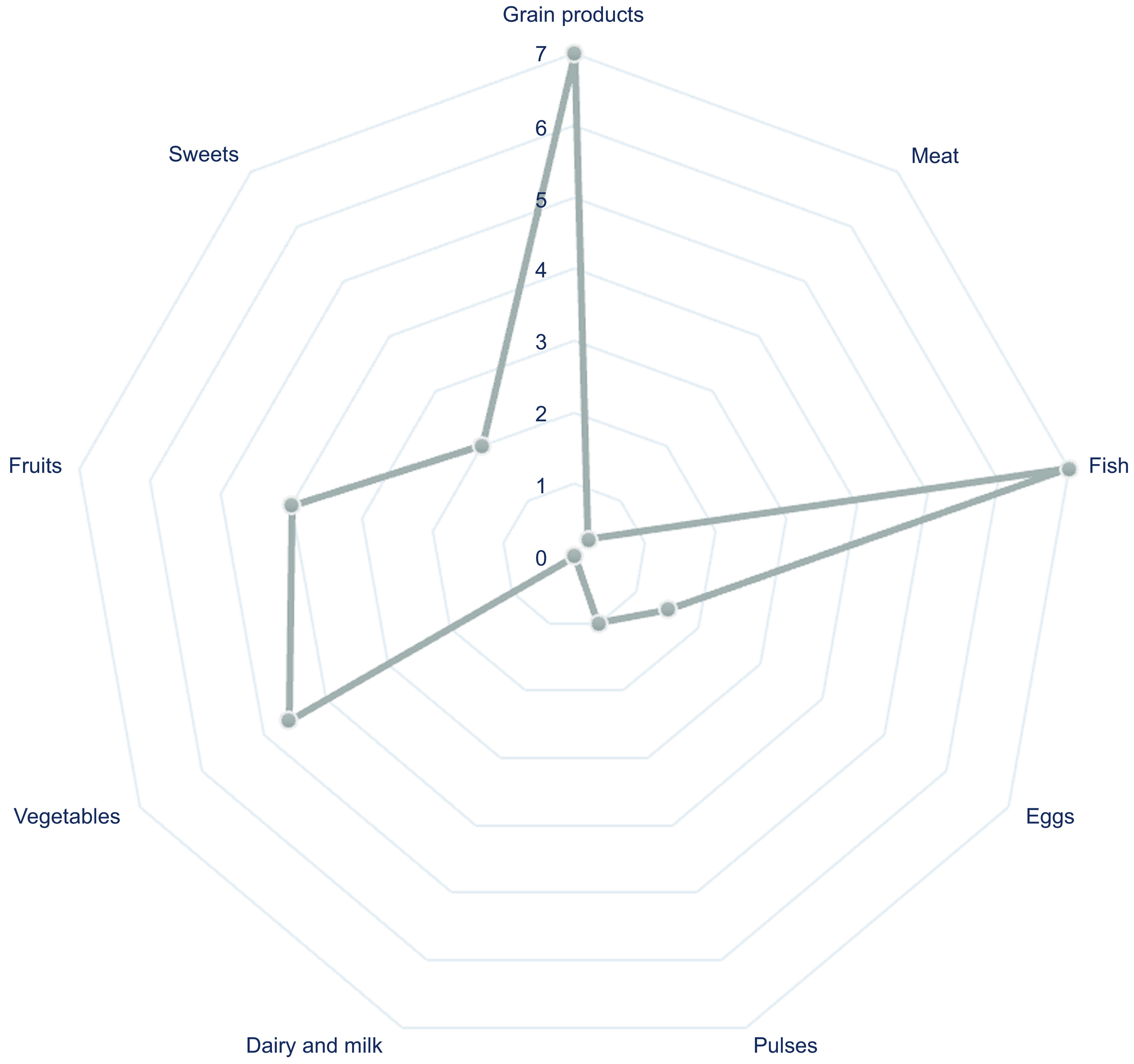
Average weekly frequency of consumption of 9 different food groups, derived from the 10 categories of the MDD-W

MDD-W results are confirmed by weekly frequency consumption. The average weekly consumption of the different food groups investigated was as follows: grain product = 7, meat = 0·3, fish = 7, eggs = 1·5, pulses = 1, dairy and milk = 0, vegetables = 4·6, fruit = 4 and sweets = 2 (Fig. 2).

The anthropometric characteristics of the participating women are presented in Table 5. The data described are related to 32 women for weight, height, triceps skinfold and mid-upper arm circumference and for 29 women for all the remaining measures, since 3 women (9·4 %) were pregnant at the time of assessment.

**Table 5:** Anthropometric characteristics of the women sample*

Variable	Values†		Min-Max
	*n*	%	
Weight (kg)			
Median	53·1		43·2–80·5
IQR	49·8–59·9		
Height (m)†			
Mean	1·6		1·4–1·6
sd	0·1		
BMI (kg/m^2^)			
Median	21·1		17·3–32·7
IQR	20·4–25·5		
Underweight, BMI < 18·5	3	10·3 %	
Normal, 18·5 ≤ BMI < 25	18	62·1 %	
Overweight, 25 ≤ BMI < 30	6	20·7 %	
Obesity, BMI ≥ 30	2	6·9 %	
Abdominal circumference (cm)†			
Mean	80·7		61·0–104·0
sd	10·2		
Waist circumference (cm)			
Median	91·5		84·0–109·0
IQR	89·0–97·0		
Mid-upper arm circumference (MUAC)			
Median	26·5		22·5–37·0
IQR	25·3–30·0		
MUAC ≤ 24 cm	6	18·8 %	
MUAC > 24 cm	26	81·2 %	
Skinfold (mm)			
Median	9·0		4·0–24·0
IQR	7·0–14·0		
Waist-to-hip ratio (WHR)			
WHR ≥ 0·85	16	55·2 %	
WHR < 0·85	13	44·8 %	
Waist-to-height ratio (WHtR)			
WHtR ≥ 0·5	15	51·7 %	
WHtR < 0·5	14	48·3 %	

Description of anthropometric measurements. The data described are related to 32 women for weight, height, triceps skinfold and mid-upper arm circumference and for 29 women for all the remaining measures.

For weight, height,

* Values are median (IQR) for continuous variables and *n* (%) for categorical variables.

† Values are mean (sd).

The median BMI was 21·1 kg/m^2^ (interquartile range: 20·4–25·5), 62·1 % had a normal weight while 27·6 % were overweight or obese. Considering the median value of waist circumference to hip circumference or height ratio, 55·2 % of the women exceeded the cut-off for waist-to-hip ratio (≥0·85) and 51·7 % of the women had waist-to-height ratio above the cut-off (≥0·5). At the same time, 81·2 % of the women had a MUAC above the cut-off point for a normal nutritional status (>24 cm).

### Children and adolescents

The mean age of the children’s sample was 7·3 ± 4 years old, with a mean age for males of 8·2 ± 4·5 years and 6·1 ± 2·8 years for girls. The mean z-scores and distribution of growth indicators used did not differ significantly between boys and girls except for the z-score of MUAC with lower values for boys (–0·9 ± 0·8) compared to girls (–0·4 ± 0·8) (*P*-value = 0·041) (Table 6).

**Table 6:** Anthropometric measurement in children’s sample by sex (*n* 36)

	Sex						*P*-value
	Boys (*n* 21)		Girls (*n* 15)		Total (*n* 36)		
	Values		Values		Values		
	Absolute frequencies	%	Absolute frequencies	%	Absolute frequencies	%	
Age (years)	max-min: 1·0–17·0		max-min: 3·0–11·0		max-min: 1·0–17·0		0·114
Mean	8·2		6·1		7·3		
sd	4·5		2·8		4·0		
Weight (kg)							
Unstandardised*							
Median	23·4		15·7		18·8		
IQR	16·5–27·5		14·1–21·5		14·1–26·6		
Z-score°							
Mean	−1·4		−1·1		−1·3		0·269†
sd	1·1		0·9		1·0		
WHO cut-off points							
Severe wasting	3	14·3 %	0	0·0 %	3	8·3 %	
Wasting	1	4·8 %	1	6·7 %	2	5·6 %	
Normal	17	81·0 %	14	93·3 %	31	86·1 %	
Height (cm)							
Unstandardised°							
Mean	121·0		111·5		117·1		
sd	24·5		17·1		22·0		
Z-score*							
Median	−1·0		−0·4		−0·9		0·344‡
IQR	−1·7 – –0·3		−1·5 – 0·2		−1·6 – –0·1		
WHO cut-off points							
Severe stunting	1	4·8 %	0	0·0 %	1	2·8 %	
Stunting	3	14·3 %	1	6·7 %	4	11·1 %	
Normal	17	81·0 %	14	93·3 %	31	86·1 %	
BMI							
Unstandardised*							
Median	14·5		14·0		14·5		
IQR	14·0–15·2		13·8–15·4		13·8–15·3		
Z-score°							
Mean	−1·3		−0·8		−1·1		0·176†
sd	1·1		1·0		1·1		
WHO cut-off points							
Severe wasting	2	9·5 %	0	0·0 %	2	5·6 %	
Wasting	2	9·5 %	1	6·7 %	3	8·3 %	
Normal	17	81·0 %	14	93·3 %	31	86·1 %	
MUAC							
Unstandardised*							
Median	17·5		17·0		17·0		
IQR	15·4–18·4		15·7–19·0		15·6–18·4		
Z-score°							
Mean	−0·9		−0·4		−0·7		0·041†
sd	0·8		0·8		0·8		
WHO cut-off points							
Moderate Negative	2	9·5 %	0	0·0 %	2	5·6 %	
Normal	19	90·5 %	15	100·0 %	34	94·4 %	

For each anthropometric measurement, three variables were reported: first represent the unstandardised value (values reported as median and interquartile range or mean and sd according to Shapiro-Wilk tests), second represent the same value standardised for age (values reported as median and interquartile range or mean and sd according Shapiro-Wilk tests), the last is a categorisation based on WHO cut-off (values reported as absolute frequencies and percentage). Reference wasting values: Severe wasting: WAZ/BAZ < –3sd; Wasting: –3sd ≤ WAZ/BAZ < –2sd; Normal: –2sd ≤ WAZ/BAZ ≤ 2sd. Reference stunting values: Severe stunting: HAZ < –3sd; Stunting: –3sd ≤ HAZ < –2sd; Normal: HAZ ≥ –2sd. The value for Moderate negative: –3sd ≤ MCAZ < –2sd; Normal: –2sd ≤ MCAZ ≤ 2sd. Statistical significance threshold: *P*-value = 0·05.

* Values reported as median and interquartile range.

Test applied:

† *t* test.

‡ Mann–Whitney *U* test.

## Discussion

The nutrition team’s objective for the cross-sectional part of the Tany Vao pilot study is to provide baseline data to design a context-specific intervention aiming to improve the nutritional condition of the population living in the rural areas of Madagascar. This manuscript presents the results related to the determinants and indicators of nutritional status, including nutritional knowledge, dietary intake, diet quality and anthropometric measurements of the study population.

Regarding nutritional knowledge, questions administered highlighted a wide gap, especially regarding attitudes to be adopted in breast-feeding, water intake and calcium sources (Table 2). This lack of education is concerning because female literacy, which includes nutritional knowledge, is strongly associated with many human development indicators, such as infant and under-5 mortality, infant and young child feeding practices and agricultural productivity^(28,29)^. Maternal higher education was associated with lower risk of stunting in children under 2 years in Indonesia and Ethiopia^(28,30)^. Also, increased maternal knowledge of infant and young child feeding was shown to decrease risk of stunting and improve child development in Indonesia^(31)^, while in Africa it reduced malnutrition when coupled with agricultural strategies, educational workshops and supplementation^(32)^ or minimum level of household resources^(33)^. The impact of mother’s nutrition knowledge on child’s nutritional status was underlined repeatedly in several African countries. In the Volta region of Ghana, mothers with higher nutritional knowledge were more likely to have well-nourished children compared to mothers with low nutritional knowledge^(34)^; similarly in Nigeria, it was associated with more height or weight-for-age z-scores^(35)^, and in Mozambique, it was associated with height^(36)^. That said, it is necessary to increase women’s nutritional knowledge to ensure not only a healthy and safe pregnancy and breast-feeding but also to welcome healthy pregnancy and to ensure healthy and safe growth for their offspring. For all these reasons, it is crucial to start the intervention as early as childbearing age and throughout the first 1000 d of the child’s life^(37)^.

Table 3 describes nutrient intake. The majority of the population (i.e. 93·1 %) has an adequate energy intake. With respect to the macronutrient consumption, protein intake is due to the large consumption of rice. This requires special attention since to reach the adequate essential amino acid profile it is fundamental to combine complementary protein sources^(38)^. When it comes to carbohydrates and sugar, remarkable is that sugars consumed are not intrinsic such as the ones found in fruits and vegetables naturally combined with fibre, vitamins and minerals, but are simple sugars (i.e. sucrose), used as an ingredient in baked foods/sweets. Their excessive consumption could contribute to an unhealthy diet, weight gain and increased risk of NCD^(39)^, exacerbated by the high saturated fat consumption^(40)^ by the 27·6 % of the population.

This is especially relevant since the mean intake of fibre, which retards sugar absorption, among other functions, is inadequately low in 93·9 % of the population, with an average intake of 12·5 g/d, where guidelines suggest a consumption of 25–28 g/d depending on the age and physiological condition (i.e. pregnancy)^(41)^.

In terms of micronutrients, vitamin A, the mean intake is lower than the recommended safe intake for all the population groups, since only 37·5 % of the population eats vitamin A-rich fruits and vegetables. Vitamin E RDA is also missed by 68·8 % of the population, probably because the group ‘nuts and seeds’ is not consumed at all. Moreover, folate, which can be found in green leafy vegetables, is not sufficiently consumed by 93·8 % of the population, even though the category of vegetables is reported to be consumed daily by 71·9 % of the population. Failure to meet RDAs of calcium, iron and zinc, must be highlighted, considering their centrality covered by those micronutrients in the correct development of the foetus and the state of maternal health. This could be due to the rare consumption of milk, dairy products, legumes, meat and poultry.

Failure to achieve the recommended nutrient intake for many micronutrients, by most of the population, is closely related to the MDD-W result (Table 4). The results are supported by another study in the Amoron’i Mania region of Madagascar, where 88 % of mothers have a dietary diversity score below 5^(11)^. This is the practical evidence of a higher risk of micronutrient-related malnutrition, since a minimum dietary diversity intake predisposes to an augmented risk of micronutrient deficiency, which is particularly harmful for women in childbearing age and pregnant women, as it might cause anaemia, neural tube defects, low birth weight and stillborn.

This condition is aggravated by the fact that the study population has excessive consumption of energy, simple sugars and salt, leading to an overweight/obesity condition and a higher risk of developing NCD^(42,43)^, depicting a double burden of malnutrition.

The fact that the majority of the population has at least adequate energy intake reflects the prevalence of women (i.e. 26 out of 29) with normal or overweight weight. The results related to women’s anthropometric measurements (Table 5) are in line with what is described in other studies conducted in Madagascar^(10)^ and other Sub-Saharan states on non-pregnant women of childbearing age^(44,45)^. However, they are not in line with the Global Nutrition Report’s projections for Madagascar in 2016, as there is a lower prevalence of underweight (10·3 % compared to the projected 13·7 %) and a higher percentage of normal weight (62·1 % compared to the projected 54·5 %) in the sample of women^(46)^. This inconsistency could be related to two factors: first, the sample size of the study population is small and therefore the results obtained cannot be interpreted as representative of the entire population; and secondly, the context of the Mitsio Islands is highly unique, therefore different from the large island of Madagascar.

All the anthropometric measures were higher than the cut-offs defined for the American and/or Caucasian populations: these findings are consistent with reports from the same population (i.e. non-pregnant women)^(44)^. The increase in mean waist-to-hip ratio and waist-to-height ratio indices is directly derived from the increase in waist circumference, while the higher arm muscle area could be explained since African women do heavier work (e.g. fieldwork) compared to that done by American women, which directly translates into greater muscle representation in their arms^(45)^. All these findings indicate the pressing need to define ethnic-specific cut-offs so as not to introduce a confounding factor in the analysis of adipose distribution, cardiovascular risk and the presence of malnutrition.

The results related to children’s anthropometric measurements (Table 6) show that most of the children sample is in a normal condition for weight, height and BMI according to the WHO cut-off points. Also, the MUAC is normal in almost all the children’s study population (94·4 %), which shows an adequate nutritional status^(20)^. The prevalence of stunting in the sample is 13·9 %, of which 2·8 % are severe conditions, which is, in general, lower than the stunting prevalence in rural areas (26·8 %), as reported by the Global Nutrition Report^(47)^. On the contrary, wasting is more prevalent in the studied population compared to the global rural prevalence found at 6·4 %^(47)^. This means that there is a coexisting condition of wasting, overnutrition and micronutrient-related malnutrition in the same household, so a triple burden of malnutrition^(47)^. This is not surprising given extreme weather conditions, economic crises, conflicts and persistent food loss and waste, which pose additional threats to the food system’s inability to provide equitable access to healthy diets for all^(48)^.

### Limitations and strengths

The authors highlighted the limitations associated with this pilot study. First, the sample size is recognised as a constraint, closely related to the intrinsic nature of the study. Secondly, the absence of specific cut-offs tailored to this population poses another limitation; this raises the urgency of identifying population-specific cut-off points. Furthermore, another lack in the literature is related to the absence of a database with the bromatological composition of the Madagascar foods. On a positive note, since the authors recognised this as a limitation, a population-specific food database was developed. This tool encompasses total energy, macronutrients and key micronutrients, forming the basis for evaluating food intake adequacy/inadequacy. Other strengths of the study are the use of the validated MDD-W and the data integrity. In fact, all research assistants underwent adequate training before engaging in data collection. As an additional strength, it is important to acknowledge the effort to have researched a complex, remote and hostile context and to provide mapping of an area never previously described.

### Conclusion

Women’s food literacy, including nutrition knowledge, is greatly related to a variety of human development indicators, including infant mortality and agricultural productivity^(49)^. This reveals the importance of increasing women’s nutrition knowledge in order to guarantee a healthy arrival to a safe pregnancy and lactation, to prevent their children from experiencing wasting and stunting during childhood. For all of these reasons, it is vital to begin intervention as early as childbearing age and continue throughout the first 1000 d of the child’s life to ensure appropriate and healthy growth^(38)^. Furthermore, increased nutritional knowledge leads to better awareness in terms of food choices, which leads to more dietary diversity and so to a lower risk of developing micronutrient-related malnutrition. Referring to a rural context, it is important to specify that nutrition knowledge is not the only aspect to work on to improve the health status of this population. Even access to food, social and economic aspects are crucial in improving the entire food system. This suggests why a multidisciplinary intervention tailored to the population and respectful of local culture, might be the winning approach.

This paper establishes the groundwork for crafting a personalised intervention based on the obtained results. The intervention is designed to enhance anthropometric parameters, nutritional knowledge and dietary diversity among the study participants, with the overarching goal of improving their nutritional conditions – a crucial dimension of the local population’s health status.

## Supporting information

Conti et al. supplementary material Conti et al. supplementary material

## Supplementary material

For supplementary material accompanying this paper visit https://doi.org/10.1017/S1368980024000259
