# An overview about nutritional status of childbearing age women, children and adolescents, living in rural areas of Madagascar: preliminary results of the Tany Vao project – CORRIGENDUM

**DOI:** 10.1017/S136898002400079X

**Published:** 2024-04-11

**Authors:** Maria Vittori Conti, Leila Itani, Alice Beretta, Edoardo Bono, Kassandra Yaghi, Asia Filosa, Cristina Monti, Hellas Cena

The authors of the above paper would like to apologise for the omission of Edoardo Bono from the author list, which has since been updated, along with the Authorship section.

Further, Fondazione Istituto Vismara De Petri Onlus was erroneously credited for partial funding instead of the correct Fondazione Peppino Vismara. This has also been updated.
