# Supplementary material for: An overview of the nutritional status of childbearing age women, children and adolescents living in a rural area of Madagascar: preliminary results of the Tany Vao project
Source: Public Health Nutr. 2024 Jan 29;27(1):e52. doi: 10.1017/S1368980024000259 (PMC10882536; doi:10.1017/S1368980024000259)
Supplement: Conti et al. supplementary material [file S1368980024000259sup001.docx]

**Table S1.** Energy, macro and micronutrients references.

|  | **Age group** |  | **References** |
| --- | --- | --- | --- |
| *Energy (kcal)* | < 18 years | 2503 kcal/day | 24,25 |
|  | 18-29 years | (14.818*BW + 486.6)*1.6 |  |
|  | 30-59 years | (8.126*BW+845.6)*1.6 |  |
|  | > 60 years | (9.082*BW+658.5) |  |
| *Total protein (g)* | < 19 years | 0.85*BW | 31 |
|  | 19-65 | 0.8*BW |  |
| *Total fat (g)* |  | 20-35% En | 26 |
| *Saturated fat (g)* |  | 10%* En | 26 |
| *Cholesterol* | > 18 years | 300 mg /die | 27,28 |
| *Cho (g)* | > 14 years | 130 g/die | 26 |
|  | Pregnant women | 170 g/die |  |
|  | Lactating women | 210 g/die |  |
| *Sugar (g)* |  | < 15% En | 29 |
| *Added sugar (g)* |  | < 10 % En | 29 |
| *Fibre (g)* | < 19 years | 26 g/die | 26 |
|  | 19-49 years | 25 g/die |  |
|  | > 49 years | 21 g/die |  |
| *Vitamin A (µg)* | < 19 years | 600 µg/die | 30 |
|  | 19-65 years | 500 µg/die |  |
|  | Pregnant women | 800 µg/die |  |
| *Beta-carotene (µg)* | < 19 years | 100.2 µg/die | 30 |
|  | 19-65 years | 83.5 µg/die |  |
|  | Pregnant women | 133.6 µg/die |  |
| *Vitamin E (mg)* | < 65 years | 15 mg/die | 30 |
|  | Pregnant women | 19 mg/die |  |
| *Vitamin C (mg)* | < 19 years | 40 mg/die | 30 |
|  | 19-65 years | 45 mg/die |  |
|  | Pregnant women | 55 mg/die |  |
| *Vitamin B12 (µg)* | < 65 years | 2.4 µg/die | 30 |
|  | Pregnant women | 2.6 µg/die |  |
| *Folate (µg)* | < 65 years | 400 µg/die | 30 |
|  | Pregnant women | 600 µg/die |  |
| *Calcium (mg)* | < 19 years | 1300 mg/die | 30 |
|  | 19-50 years | 1000 mg/die |  |
|  | 51-65 years | 1300 mg/die |  |
|  | Pregnant women | 1200 mg/die |  |
| *Iron (mg)* | < 19 years | 15 mg/die | 3 |
|  | 19-50 years | 8 mg/die |  |
|  | 51-65 years | 18 mg/die |  |
|  | Pregnant women | 27 mg/die |  |
| *Zinc (mg)* | < 19 years | 14.4 mg/die | 30 |
|  | 19-56 years | 9.8 mg/die |  |
|  | Pregnant women | 11 mg/die |  |
| *Magnesium (mg)* |  | 220 mg/die | 3 |
| *Sodium (mg)* |  | 2000 mg/die | 30,31 |
